# Brain structure in pediatric Tourette syndrome

**DOI:** 10.1038/mp.2016.194

**Published:** 2016-10-25

**Authors:** D J Greene, A C Williams III, J M Koller, B L Schlaggar, K J Black, and The Tourette Association of America Neuroimaging Consortium

**Affiliations:** 1 Department of Psychiatry, Washington University School of Medicine, St Louis, MO USA; 2 Department of Radiology, Washington University School of Medicine, St Louis, MO USA; 3 Washington University School of Medicine, St Louis, MO USA; 4 Department of Neurology, Washington University School of Medicine, St Louis, MO USA; 5 Department of Neuroscience, Washington University School of Medicine, St Louis, MO USA; 6 Department of Pediatrics, Washington University School of Medicine, St Louis, MO USA

**Keywords:** Neuroscience, Psychiatric disorders

## Abstract

Previous studies of brain structure in Tourette syndrome (TS) have produced mixed results, and most had modest sample sizes. In the present multicenter study, we used structural magnetic resonance imaging (MRI) to compare 103 children and adolescents with TS to a well-matched group of 103 children without tics. We applied voxel-based morphometry methods to test gray matter (GM) and white matter (WM) volume differences between diagnostic groups, accounting for MRI scanner and sequence, age, sex and total GM+WM volume. The TS group demonstrated lower WM volume bilaterally in orbital and medial prefrontal cortex, and greater GM volume in posterior thalamus, hypothalamus and midbrain. These results demonstrate evidence for abnormal brain structure in children and youth with TS, consistent with and extending previous findings, and they point to new target regions and avenues of study in TS. For example, as orbital cortex is reciprocally connected with hypothalamus, structural abnormalities in these regions may relate to abnormal decision making, reinforcement learning or somatic processing in TS.

**Supplementary information:**

The online version of this article (doi:10.1038/mp.2016.194) contains supplementary material, which is available to authorized users.

## Introduction

Tourette syndrome (TS) is a developmental disorder of the central nervous system defined by the chronic presence of primary motor and vocal tics.^1^ Tics are repeated, nonrhythmic, unwanted but usually suppressible movements or vocalizations.^2^ TS usually involves one or more additional feature, most often obsessions, compulsions, distractibility or impulsivity.^3^ A clear neurobiological explanation for TS is not yet available, but research has provided many relevant clues.^4, 5^

A number of studies have now examined the structure of the living brain in TS and have found significant changes in various brain regions compared with tic-free healthy control subjects.^6, 7^ The largest studies were reported by Peterson *et al.*, with over 100 children and adults with TS and a similar number of control subjects. However, substantial questions remain about the structural anatomy of the brain in TS because methods and results have varied widely across studies, and because most studies were from small samples. A multicenter collaborative approach to brain imaging in TS might address these and other concerns.

Here we believe we report the first analysis from such a collaboration, the Tourette Association of America Neuroimaging Consortium, applying structural magnetic resonance imaging (MRI) to large, well-matched groups of children and adolescents with and without TS.

## Materials and methods

This study was approved by the Washington University Human Research Protection Office (IRB), protocol # 201108220. Most MRI and clinical information were originally collected under different IRB protocols (independent of this study) at the four imaging sites: Washington University School of Medicine (WUSM), New York University (NYU), Kennedy Krieger Institute at Johns Hopkins University School of Medicine (KKI), University of California, Los Angeles (UCLA). Subjects’ guardians gave informed consent for participation in the original studies. Herein we call these ‘legacy’ data. The WUSM, KKI and UCLA sites enrolled additional new subjects specifically for this study. The transmission of any human subjects data to the consortium was approved by each site’s respective IRB. Some data were provided anonymously to the consortium under code-sharing agreements.

Imaging data were stripped of personal identifiers such as name and date of birth and archived at the Central Neuroimaging Data Archive (CNDA) hosted at https://cnda.wustl.edu.^8^ REDCap electronic data capture tools hosted at Washington University were used to manage the clinical data collected at WUSM.^9^

### Subjects

**Figure 1:**
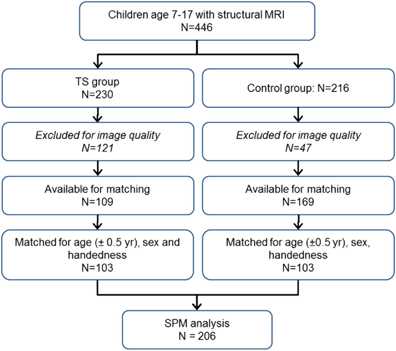
Subject flow diagram. MRI, magnetic resonance imaging; TS, Tourette syndrome. PowerPoint slide

Existing and newly acquired T1-weighted MPRAGE images were collected in 2007–2014 from over 400 children of age 7–17 years, including 230 with a chronic tic disorder (DSM-IV-TR TS or chronic tic disorder). Authors ACW, DJG or KJB visually reviewed each structural MRI and excluded images with any visible artifact in the brain; KJB was the final arbiter and was blind to diagnosis at this review. After excluding scans with visible artifact, MPRAGE images were available from 109 TS and 169 control subjects of age 7–17 years. Of the 109 TS subjects, 103 could be matched one to one with a control subject for age (within 0.5 years), sex and handedness (Figure 1).

MPRAGE (3D T1-weighted) data were acquired on several magnetic resonance (MR) scanners with varying parameters. The most common structural image protocol was an MPRAGE with total scanning time 6–10 min and voxel size 1.0–1.25 mm^3^. In all, eight different scanner/sequence combinations were used to acquire the images (Supplementary Table 1).

### Image processing

If a subject had more than one MPRAGE image of adequate quality, these images were averaged after mutual rigid-body alignment using a validated method.^10^ All subsequent image analyses were performed with SPM software v.12b using the method of J. Ashburner.^11, 12^

Each subject’s image was nonlinearly normalized to Montreal Neurological Institute space, and the atlas-aligned images were averaged to create an MPRAGE template specific to this study (https://irc.cchmc.org/software/tom.php).^13^ For each subject, segmented images were created to reflect the probability that each voxel was composed of gray matter (GM), white matter (WM) or cerebrospinal fluid. This computation used a Bayesian approach, with prior probabilities established by population templates for GM, WM and cerebrospinal fluid to inform interpretation of the subject’s MR signal at each voxel. Alignment and segmentation were then refined by tissue-specific realignment.^14^ The tissue density images were multiplied by the local volume in the subject image corresponding to each voxel in the atlas template to produce images showing at each atlas voxel that subject’s GM, WM and cerebrospinal fluid volume contributing to that voxel. The GM volume image from one subject is shown in Supplementary Figure 1. A three-dimensional Gaussian filter (full-width at half-maximum 6 mm) was applied to the GM and WM images and the smoothed images were submitted to SPM analysis.

### Analysis

SPM software v. 12b (http://www.fil.ion.ucl.ac.uk/spm/software/) computed at each voxel a general linear model with dependent variable GM volume; factors diagnostic group, MRI scanner and sequence (Supplementary Table 1), and sex; age at scan as a covariate; and interactions of group × sex and sex × age. Proportional scaling by each subject’s total GM+WM volume corrected for global brain volume. The GM analysis was limited to voxels at which GM concentration was >20%. The WM analysis used the same methods.

One-tailed contrasts were used to generate *t* images comparing TS and control groups, without assuming equal variance. Statistical significance was determined by the volume of clusters defined by contiguous voxels with |*t*|>3.0, corrected to a false discovery rate of 5%. Peak voxel locations in Montreal Neurological Institute space were transformed to Talairach atlas coordinates using MNI2TAL (http://bioimagesuite.yale.edu/mni2tal).^15^

Total GM and total WM were modeled similarly, that is, with diagnostic group, MRI sequence and sex as factors, age at scan as a covariate and interactions of group × sex and sex × age, but of course omitting the global volume correction, using R statistical software v. 3.1.2.^16^

Secondary analyses focused on the key findings from the SPM analyses. For each significant cluster from the SPM analyses of GM, the sum of each subject’s GM volume over all voxels in that cluster was corrected for the subject’s total brain volume (GM+WM) by division. The same was done for the significant WM clusters. These relative cluster volumes for each subject were the dependent variables to test for effects of scanner and sequence, age, attention deficit hyperactivity disorder (ADHD), intelligence quotient (IQ), Yale Global Tic Severity Scale total tic score (YGTSS) and medication status, either in the entire TS group, in a subset that had complete data for the analysis at hand, or in the subjects scanned with sequence 3 from Supplementary Table 1 (the largest group of subjects scanned using the same scanner and sequence).

## Results

### Subjects

Demographic and illness variables are summarized in Table 1.

**Table 1:** Demographics and illness variables

	*TS group*	*Control group*	P
*N*, total→used	230→103	216→103	1.00
*N* by site, total→used			<0.001^a^
WUSTL	141→78	156→70	
UCLA	51→13	0→0	
NYU	25→8	38→21	
KKI	13→4	22→12	
Age (years, mean±s.d.)^b^	11.9±2.1	11.9±2.1	0.96
Sex (M: F)	81: 22	81: 22	1.00
Handedness (# right-handed)	103	103	1.00
YGTSS Total Tic Score	18.1±8.3 (*N*=91)^c^	n/a	—
ADHD clinical diagnosis	55% (43 out of 78)^c^	—^d^	—
CY-BOCS score (mean±s.d.)	5.3±6.8 (*N*=65)^c^	—^d^	—
OCD clinical diagnosis	47% (15 out of 32)^c^, ^e^	—^d^	—
Medication status^f^	Adrenergic agonists (*N*=25)	n/a	—
	Stimulants (*N*=21)		
	Antidepressants (*N*=7)		
	Antipsychotics (*N*=5)		
	Norepinephrine reuptake inhibitors (*N*=2)		
	None of the above (*N*=36)		
IQ (mean±s.d.)	108±13 (*N*=80)^c^	119±12 (*N*=39)^c^	< 0.001^g^

Abbreviations: ADHD, attention deficit hyperactivity disorder; CY-BOCS, Children's Yale-Brown Obsessive-Compulsive Scale; IQ, intelligence quotient; KKI, Kennedy Krieger Institute; NYU, New York University; OCD, obsessive compulsive disoreder; UCLA, University of California, Los Angeles; TS, Tourette syndrome; WUSTL, Washington University in St. Louis; YGTSS, Yale Global Tic Severity Scale total tic score.

^a^*χ*^2^=56.4, 3 df.

^b^Starting with this row, data describe only the final 206 subjects.

^c^Not available for all subjects.

^d^Not available for most control subjects.

^e^45% (35 out of 78) had CY-BOCS score>0.

^f^Medication information available for 75 TS subjects.

^g^*t*=4.53, 81.9 df (two-sided *t*-test, unequal variance, Welch df modification).

### Global volumes

Total GM volume was significantly correlated with age (*P*<0.001), but no other factors, covariates or interactions were significant in the analysis of total GM or total WM (*P*⩾0.30).

### Regional differences in WM volume in TS

**Figure 2:**
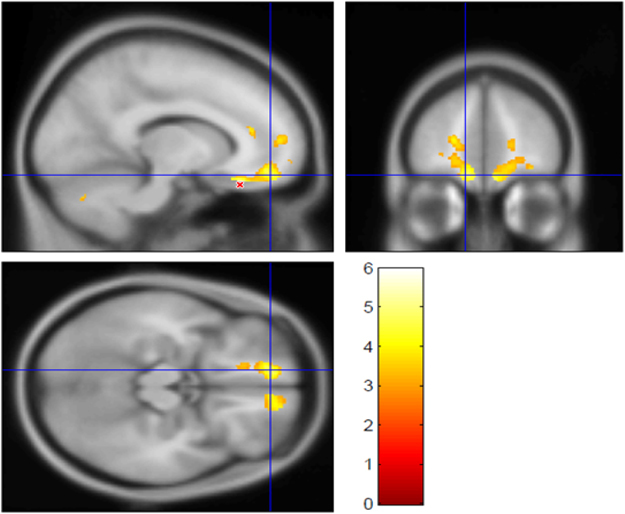
The largest cluster from the contrast showing where WM volume is lower in TS than in the control group (5.2 ml, *p*_FDR_ =0.001; see Table 2). The *t* statistic is shown in color (thresholded at *t*⩾3.0), laid over the average MP-RAGE image from the entire sample (in grayscale). The crosshairs show (−12, 49.5, −16.5)_MNI_, left medial orbital gyrus, BA11. The peak *t-*value from this contrast, *t*_193_=5.95, is at (−13.5, 31.5, −22.5)_MNI_ in left medial orbital gyrus, BA13, near the red ‘X’ in the sagittal image. Supplementary Figure 2 shows the other significant cluster from this contrast, the homologous area on the right side of the brain. FDR, false discovery rate; MNI, Montreal Neurological Institute template brain coordinates; TS, Tourette syndrome; WM, white matter. PowerPoint slide

Table 2 summarizes the VBM results. Two fairly symmetric WM clusters showed lower volume in TS, each corrected *P*=0.001, located in WM deep to orbital and medial prefrontal cortex (Figure 2, Supplementary Figure 2).

**Table 2:** VBM results

*p*_*FDR*_	*Volume (ml)*	*Peak t*	*Peak (MNI)*			*Peak (TT)*			*Description*
			*x*	*y*	*z*	*x*	*y*	*z*	
*TS<control: white matter*									
0.001	5.2	5.95	−13.5	31.5	−22.5	−14	27	−20	L medial orbital gyrus, BA13^a^
		4.83	−12.4	9.5	−16.5	−12	44	−17	L medial orbital gyrus, BA11
		4.75	−15	22.5	−21	−15	18	−17	L medial orbital gyrus, BA11
		4.07	−19.5	51	9	−19	47	7	L medial frontal gyrus, BA10
		3.93	−19.5	43.5	−12	−20	39	−11	L OFPFC, BA11
		3.91	−13.5	36	15	−14	33	14	WM deep to L BA32
		3.9	−15	55.5	9	−15	52	7	WM in L anterior PFC
		3.78	−16.5	46.5	3	−16	43	2	WM in L anterior PFC
		3.59	−18	51	−7.5	−18	46	−8	WM deep to L BA 11/12^a^
		3.54	−27	55.5	−10.5	−27	51	−10	L OFPFC, BA12^a^
		3.19	−22.5	36	21	−21	34	20	WM, middle of PFC
0.001	4.6	4.6	7.5	49.5	−19.5	6	44	−20	WM deep to R BA11
		4.45	18	42	3	17	38	3	WM deep to R anterior cingulate, BA32
		4.03	16.5	55.5	−15	15	50	−15	R BA11
		4.02	15	63	7.5	14	58	5	R BA10, medial frontal gyrus
		3.93	21	43.5	−7.5	20	38	−5	WM deep to R BA47/12^a^
		3.69	7.5	58.5	−13.5	6	52	−14	R medial PFC, BA10^a^
		3.59	30	49.5	−12	29	44	−10	WM deep to R BA47/12^a^
		3.24	13.5	42	−19.5	12	36	−18	R olfactory sulcus, BA11m/l^a^
0.196	1.2	3.98	30	−10.5	4.5	29	−11	7	R posterior putamen
		3.46	31.5	−13.5	−4.5	30	−14	0	R posterior putamen
		3.41	34.5	−15	12	33	−15	14	R posterior insula
		3.39	28.5	0	9	27	−1	11	R putamen
0.223	1.1	3.89	−28.5	−10.5	4.5	−27	−11	6	L posterior putamen
		3.5	−33	−6	13.5	−32	−7	14	L posterior insula
		3.46	−25.5	1.5	10.5	−24	−1	11	L putamen
*TS>control: gray matter*									
0.001	4.4	4.62	−13.5	−30	9	−12	−30	11	L thalamus, pulvinar n.
		4.26	−15	−28.5	−4.5	−14	−29	0	L thalamus
		3.75	0	−33	−4.5	0	−33	0	Dorsal edge of midbrain
		3.46	0	−34.5	−13.5	0	−35	−7	Dorsal edge of pons / midbrain
0.011	2.7	4.06	9	−3	−16.5	8	−5	−11	Ventral edge of basal forebrain / midbrain
		3.93	−1.5	−6	−7.5	−2	−8	−3	L hypothalamus
		3.76	0	−15	−10.5	0	−16	−5	Ventral midbrain, near supramammillary commissure
0.07	1.6	4.13	16.5	−28.5	−4.5	15	−29	0	R thalamus, posterior edge
		3.9	12	−30	12	12	−30	14	R thalamus, posterior edge

Abbreviations: BA, Brodmann area; FDR, false discovery rate; L, left hemisphere; MNI, Montreal Neurological Institute template brain coordinates; OFPFC, orbitofrontal prefrontal cortex; PFC, prefrontal cortex; R, right hemisphere; TS, Tourette syndrome; TT, Talairach and Tournoux atlas coordinates; WM, white matter.

*p*_FDR_, FDR corrected *p* value for a suprathreshold cluster of this size in the *t* image. For each local maximum (peak) in the cluster, the table lists the *t* statistic at that voxel (193 df) and the atlas coordinates of that voxel’s location. TS >control: white matter, no significant clusters. TS<control: gray matter, no significant clusters.

^a^Description taken from (Öngür and Price^50^).

Two additional symmetric clusters of decreased WM volume are of interest, though they did not remain statistically significant after multiple comparisons correction (each *P*=0.2). These clusters include parts of posterior putamen and insula bilaterally (Supplementary Figure 3).

### Regional differences in GM volume in TS

**Figure 3:**
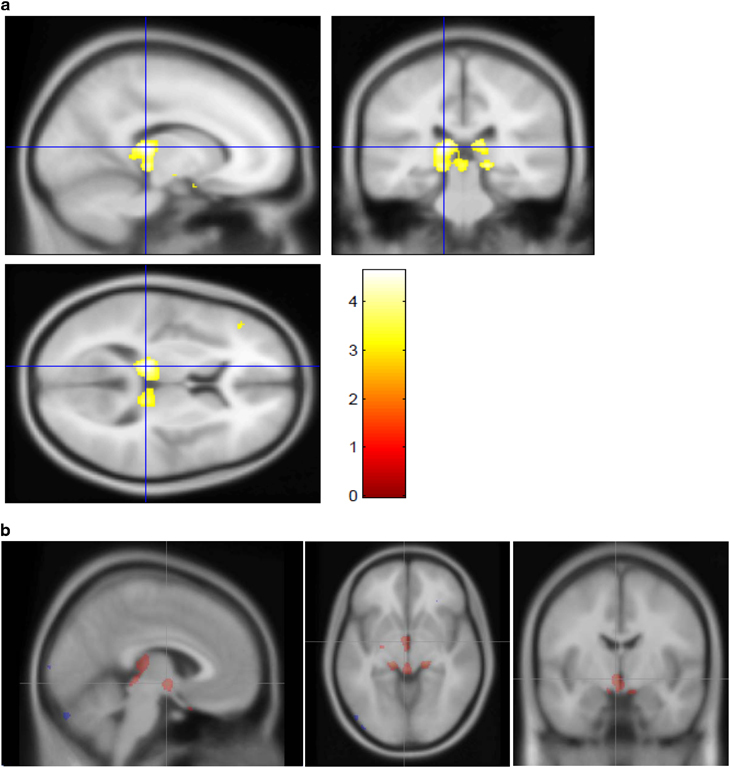
Largest clusters showing greater GM volume in TS compared with controls. (**a**) Largest cluster from GM>control contrast, in left pulvinar nucleus of thalamus (see Table 2 and legend to Figure 2). (**b**) The second largest cluster from the GM>control contrast, with the crosshairs at (4, 6, −6)_MNI_ in hypothalamus. In this figure, all voxels with *t*⩾3.0 are highlighted in color to better visualize the underlying anatomy. GM, gray matter; MNI, Montreal Neurological Institute template brain coordinates; TS, Tourette syndrome. PowerPoint slide

Two clusters showed statistically significant increased GM volume in TS after correction for multiple comparisons (Table 2). The largest suprathreshold cluster had peak *t*-value=4.62 (193 d.f.) in the pulvinar nucleus of the left thalamus (Figure 3a). A homologous cluster in the right pulvinar was below the significance threshold (corrected *P*=0.07). The second largest cluster (corrected *P*=0.011) included the hypothalamus bilaterally and the ventral midbrain (Figure 3b).

### Secondary analysis: scanner and MR sequence

The statistical model included a factor to account for different scanners or MR sequences, but such statistical control may be imperfect. Accordingly, we checked whether the findings from the overall group would still be present if the different-scanner concern were eliminated. One site acquired images from 46 TS subjects and 27 control subjects on one scanner using the same sequence. For the left thalamus SPM cluster, for instance, the question is whether GM volume was higher in TS, as it was in the overall analysis, in these subjects who were all scanned on the same scanner with the same MR sequence. This question was tested using analysis of covariances (ANCOVAs) with relative GM volume in the SPM cluster as the dependent variable, diagnosis and sex as factors, age as a covariate and interactions of sex with diagnosis and age. As in the full SPM analysis, this cluster was larger in TS (Supplementary Figure 4a, diagnosis factor *P*=0.004), as was the hypothalamus GM cluster (Supplementary Figure 4b, diagnosis *P*<0.001, with a significant diagnosis x sex interaction *P*=0.009). The orbitofrontal cortex (OFC) WM clusters were smaller in TS (Supplementary Figure 4c,d, left WM diagnosis *P*<0.001, right *P*<0.001).

### Secondary analysis: age, tic severity, IQ, comorbidity and medication status

We investigated whether or not differences in regional volume depended upon age, using ANCOVA for each of the significant GM and WM clusters, with relative volume as the dependent variable, diagnosis as a factor, age as a covariate, and the diagnosis × age interaction. The main effect of age was significant, with WM volume increasing with age (left OFC *P*<0.001, right OFC *P*<0.001) and GM volume decreasing with age (left thalamus *P*=0.03, hypothalamus *P*=0.02) regardless of diagnosis. No diagnosis × age interactions were significant (all *p’s* >0.3), indicating similar diagnosis effects across the age range.

Past-week tic severity as measured by the YGTSS^17^ was available for 91 out of the 103 TS subjects. In a multiple regression analysis for the relative volume in each significant cluster, we modeled YGTSS and age as factors and their interaction. The YGTSS effect and the YGTSS × age interaction were not significant in any of the models (all *p*’s >0.6), suggesting our results reflected diagnosis rather than cross-sectional tic severity.

We had IQ estimates for all but one subject from the single-sequence group discussed in the previous section. IQ differed between diagnostic groups (TS 107.5±11.9, control 117.8±13.1, *P*<0.002, unpaired *t-*test), so we checked whether IQ explained any of the primary group differences by modeling relative cluster volume by ANCOVA with sex as a factor, age and IQ as covariates, and all interactions. Neither IQ nor interactions with IQ were significant for any of the four significant clusters (*p* for IQ was 0.08 for right OFC WM, 0.27 for left OFC WM, 0.10 for left thalamus and 0.51 for hypothalamus).

ADHD was recorded for all TS subjects in that same subgroup. In an ANCOVA with sex and ADHD diagnosis as factors, age as a covariate, and all interactions, neither ADHD nor interactions with ADHD were significant for any of the four clusters. Obsessive compulsive disorder (OCD) diagnosis was not recorded in this subgroup, but as a loose proxy we dichotomized TS subjects based on OCD symptom severity (CY-BOCS scores, zero vs greater than zero). This OCD factor was not significant, nor were any interactions with this factor.

Medication status was available for 75 out of the 103 TS subjects (Table 1). Of these subjects, 39 were taking psychoactive medications and 36 were not. Since the number of subjects on any one medication was relatively small, but about half were unmedicated, we ran ANCOVAs with medication status (medicated vs unmedicated) as a factor, age as a covariate, and their interaction. There was no significant main effect of medication status or its interaction with age (*p’s* >0.3 in all four clusters).

## Discussion

Here we present the largest study of brain structure ever reported in children with TS. We matched control subjects strictly for age, sex and handedness, and the statistical analysis used conservative methods to minimize type I error. Our main findings were that the TS group had lower WM volume than the control group deep to orbital and medial prefrontal cortex, and greater GM volume in the posterior thalamus and hypothalamus.

### Lower WM volume in prefrontal cortex in TS

The finding of decreased WM in orbital cortex is consistent with several previous TS studies that showed decreased GM volumes in this region. Studies of adults with TS found reduced GM volume in OFC,^18^ a negative correlation between OFC GM volume and tic severity,^19^ and cortical thinning in OFC.^18, 20^ A study of adolescents and adults with TS (10–25 years) found increased cortical thinning with age in the right OFC compared with controls.^21^ A study that focused on WM identified 10 tracts with decreased WM integrity (scaled fractional anisotropy) in unmedicated adults with TS.^22^ Four out of the 10 tracts involved the OFC, connecting OFC with pre-SMA, ventral premotor cortex, primary motor cortex, and supplementary motor cortex. Thus, there is converging evidence for altered OFC gray and WM in adults with TS. Since TS is a developmental disorder; however, it is difficult to make direct comparisons between our results in children and previous findings in adults. Investigations of OFC volume in *children* with TS have been much more limited. In an earlier, large structural MRI study in TS, sub-analyses of child subjects demonstrated decreased volume (including GM and WM) in a predefined orbital frontal area.^23^ Our results suggest that this decreased OFC volume may be attributable to WM, and lead to questions about developmental changes in OFC in TS.

The orbitofrontal regions identified in the present study have been linked to a wide range of functional processes, yet many roles commonly attributed to OFC have been called into question.^24^ There is evidence showing that the OFC codes economic value, having a key role in decision making and reward.^25, 26, 27^ Specifically, the medial OFC, where we found reduced WM volume, is involved in the weighing of options that leads to decisions. Economic decision making has not been well-investigated in TS, but a small number of studies have examined reinforcement learning,^28^ showing evidence specifically for impaired negative reinforcement learning in youth and adults with TS. Perhaps this impairment is a functional consequence of reduced WM volume in the OFC, but such a hypothesis deserves further study.

Alternatively, altered OFC may be involved in the sensory aspects of tics. Most TS patients report that tics are often responses to uncomfortable internal sensations, like a tickle in the throat before a cough; some experts conclude that these premonitory sensations may be the primary phenomenon rather than the observed tics.^29^ Premonitory sensations in TS are correlated with sensory hypersensitivity^30^ and interoceptive awareness,^31^ yet peripheral sensation is normal,^32, 33^ so any sensory abnormality must be central. Since the OFC receives input from most sensory systems and projects to regions involved in visceral function,^34^ abnormal WM connections with OFC fit well with a sensory model of TS.

The clusters of decreased WM volume also extended to pregenual WM and WM deep to medial frontal gyrus (BA 10). A previous study that examined WM integrity in men with TS found that greater current tic severity was associated with decreased WM fractional anisotropy deep to superior frontal gyrus.^35^ While consistent with our results, this previous finding was in adults and the specific location of the region was 10mm superior to the BA 10 peak in the current study.

### Putamen

The paired clusters of decreased WM volume in posterior putamen are interesting given the posterior putamen’s prominent role in movement. Previous studies in TS have yielded mixed results regarding putamen volume, mostly focusing on GM. Although some found evidence for smaller putamen in children and adults with TS,^36, 37^ others have shown increased volume in children with TS.^38, 39^ Putaminal WM has also been implicated, as apparent diffusion coefficient in the putamen was highly correlated with tic severity in unmedicated men with TS,^35^ though the most significant voxels in that study were 16–17 mm anterior and inferior from the peaks reported here. However, the clusters in the present study were not significant after correction for multiple corrections, and a WM difference might be more easily interpreted as referring to the external capsule or extreme capsule than to the putamen itself.

### Other

Several previous TS studies in adults and children found larger volume or reduced measures of WM integrity in the corpus callosum,^18, 40, 41, 42, 43^ but otherwise, previous WM findings in TS have been variable.^6^

### Greater GM volume in pulvinar nucleus, midbrain and hypothalamus in TS

#### Pulvinar

Several imaging studies have examined thalamic volume in TS.^6^ The largest of these found increased total thalamic volume in children and adults with TS (~5%), with outward deformation (bulges) compared with thalamic shape in control subjects.^44^ The most prominent differences were found on the ventral, lateral and posterior surfaces, corresponding to several motor nuclei and the pulvinar. Thus, that study’s results are quite consistent with the present finding of greater GM volume in the pulvinar in a large group of children and adolescents. Its authors posit several possible explanations for enlargement in these thalamic regions, including hyperactive motor circuitry, compensatory mechanisms derived from years of attempting to control tics, or secondary GM changes in the face of WM alterations.

The medial pulvinar nucleus is widely connected to cortex, including prefrontal, orbital and cingulate cortical areas; the lateral pulvinar projects to parietal, temporal and extrastriate regions; and the inferior pulvinar has bidirectional connections with visual cortex.^45, 46^ Given these widespread projections and innervations, we speculate that increased GM volume in TS may relate to multisensory integration in the thalamus, or to the linking of sensory input to cognitive-, motivational- and movement-related areas of cortex. As noted above, higher-order sensory processing has been hypothesized to be important in TS, and there is evidence for a role of the pulvinar nucleus in spatial attention and attention to salient stimuli.^47, 48^

#### Midbrain

Part of the thalamus GM cluster includes dorsal midbrain. Interestingly, a VBM study of 31 adult patients and 31 controls also identified a significant increase in GM volume in midbrain,^49^ though that statistical peak was inferior and anterior to the one identified in the present study.

#### Hypothalamus

One cluster of increased GM volume included hypothalamus. We are not aware of previous studies linking TS to this structure. The hypothalamus does receive inhibitory innervation from the ventromedial OFC via the central nucleus of the amygdala.^50^ This anatomical connection is intriguing given the OFC WM changes in this study, as it has been posited that hypothalamic projections to OFC may be involved in reinforcement^26^ and reward signals.^51^ Future work may study the hypothalamus in TS more specifically.

### Comparison to ADHD and OCD

Given the high comorbidity rates of ADHD and OCD in TS,^52^ it is worth comparing our results to previous findings in these conditions. Large volumetric studies and meta-analyses of VBM data have demonstrated reduced striatal volume in children with ADHD that normalized with increasing age.^53, 54, 55, 56^ The present study found only a trend for reduced WM putamen volume in TS. There is also evidence for decreased thalamic volume in ADHD,^57, 58, 59^ whereas we found increased volume in TS. Larger thalamic volumes *have* been reported in OCD using volume-of-interest^60^ and VBM approaches;^61^ the VBM study also found increased GM volume in bilateral hypothalamus.

As for the OFC, a recent large study in 307 children and adults with ADHD found lower GM volume in several frontal regions, including OFC.^62^ However, a number of ADHD studies found alterations in other frontal regions, but not OFC.^63, 64^ A meta-analysis examining both ADHD and OCD found reduced GM volume in ventromedial OFC in both groups.^56^ OFC alterations are commonly reported in OCD, yet the specific results have been quite mixed, including both decreased^60^ and increased volume,^61, 65^ as well as more complex lateralized results.^65, 66^ Of course, our OFC findings were specific to WM volume, making direct comparisons difficult.

Overall, there are more similarities between our results and those in OCD than in ADHD, perhaps consistent with the recent demonstration that TS shares greater genetic variance with OCD than with ADHD.^67^ Nevertheless, TS with comorbid OCD may be distinct from OCD without tics.^68^ In any case, ADHD and OCD are unlikely to explain the results in the present sample given null results in the secondary analyses based on ADHD diagnosis and current OCD severity. This conclusion should be confirmed in a sample with prospective, systematic psychiatric diagnosis.

### The dog that did not bark in the night

A word is due about previous volumetric findings that were not replicated here. The most notable is decreased caudate volume in TS reported by Peterson *et al.*^36^ in a study of 154 children and adults with TS and 130 controls (including a total of 173 children), and by two other groups.^19, 35, 69, 70, 71^ Smaller caudate volume in childhood predicted worse tic severity in young adulthood, showing that decreased caudate volume could not be just a consequence or adaptation of the brain to tics.^37^ Conceivably our caudate non-finding may reflect type II error.

On the other hand, several other studies did not find significantly smaller caudate in TS (reviewed in Williams *et al.*^72^), the largest of which included 49 boys with TS and 42 controls.^39^ The present study adds to these null findings, and has particular merit due to its large sample, exclusion of adults, and use of one-to-one age and sex matching in addition to statistical accounting for linear effects of age. Furthermore, the caudate is a relatively small structure, surrounded by WM and cerebrospinal fluid, and hence especially susceptible to partial volume effects and, presumably, to the artifactual reduction in volume with frequent small-amplitude head movements demonstrated with other techniques.^73, 74^

### Limitations

The most important limitation is the use of different scanners, different sequences and possibly different recruitment sources or diagnostic methods across sites. However, the results in the largest single-sequence subgroup suggest strongly that the key findings are not driven by differences in scanner, sequence or site.

A second limitation is that phenotypic data are limited for many of the ‘legacy’ subjects. For instance, history of phonic tics is missing for some TS subjects, and for many subjects we have limited information on comorbid diagnosis. In the available data our key findings are not significantly linked to IQ, ADHD, OCD or medication status, but of course future studies will benefit from adequate prospective assessment of all these variables.

Recently, small head movement not detected by visual inspection of MR images has been shown to artifactually lower GM volume in VBM analyses, presumably by a mechanism similar to partial volume effect.^73, 74^ Fortunately, group differences in residual head movement cannot easily explain the decreased WM volume or the increased GM identified in this study, since the TS group would be expected to show more head movement.

### Future directions

Here, we identify several brain regions that can serve as new targets for further study. A prospective study design with additional clinical information can test whether the posterior thalamic finding in fact relates to sensory symptoms in TS, whether the OFC finding relates to decision making or reinforcement learning in TS, and to what extent the severity of tics and comorbid symptoms^52^ explain these findings. Future structural imaging studies can help elucidate at what age the regional differences in GM and WM volume in TS first manifest and whether they persist into adulthood, helping to clarify whether these volumetric differences represent failures of maturation or alterations after a period of normal development.

Studies with different methodology will be required to elucidate the mechanism responsible for the volumetric abnormalities. Postmortem studies in TS have not typically focused on the regions identified here.^75^ Thus it is not clear whether, for example, increased GM volume in posterior thalamus reflects increased neuronal cell number, glial cell number, neuropil (for example, deficient pruning) or increased water content. On the other hand, this reflects a potential strength of the present study: an unbiased, whole-brain analysis identified regions of brain that have hardly been studied at a cellular level in TS.

## Supplementary information

Supplementary Figure 1 (JPG 172 kb)

Supplementary Figure 2 (JPG 225 kb)

Supplementary Figure 3 (JPG 263 kb)

Supplementary Figure 4 (JPG 1086 kb)

Supplementary Table 1 (DOCX 92 kb)

## PowerPoint slides

PowerPoint slide for Fig. 1
PowerPoint slide for Fig. 2
PowerPoint slide for Fig. 3
