# Supplementary figures and images for: Brain structure in pediatric Tourette syndrome
Source: Mol Psychiatry. 2016 Oct 25;22(7):972–80. doi: 10.1038/mp.2016.194 (PMC5405013; doi:10.1038/mp.2016.194)

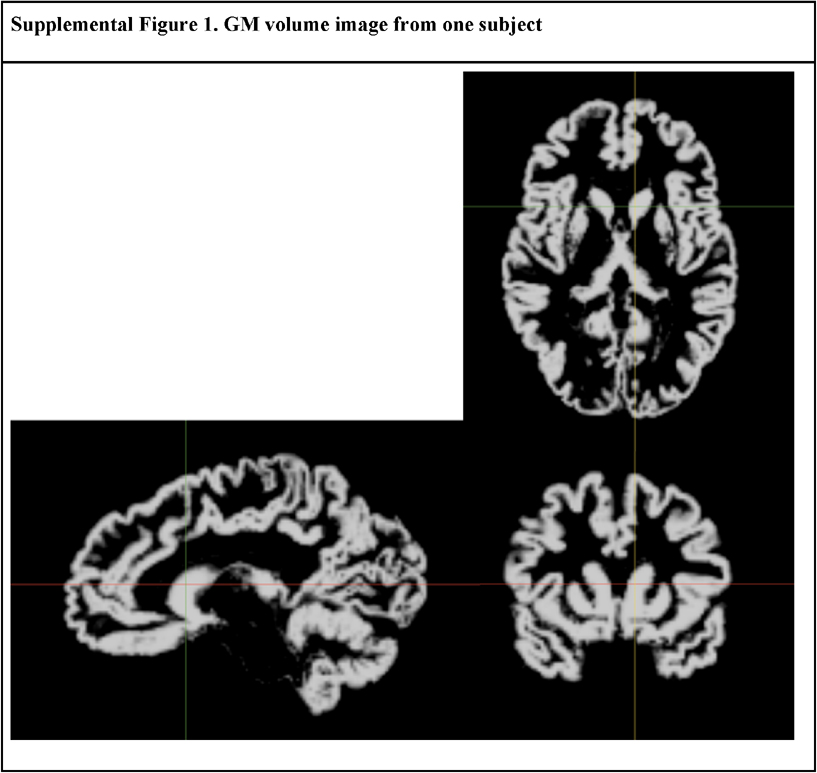

Supplement: Supplementary file 1 — Supplementary Figure 1 (JPG 172 kb) [file 41380_2017_BFmp2016194_MOESM362_ESM.jpg]

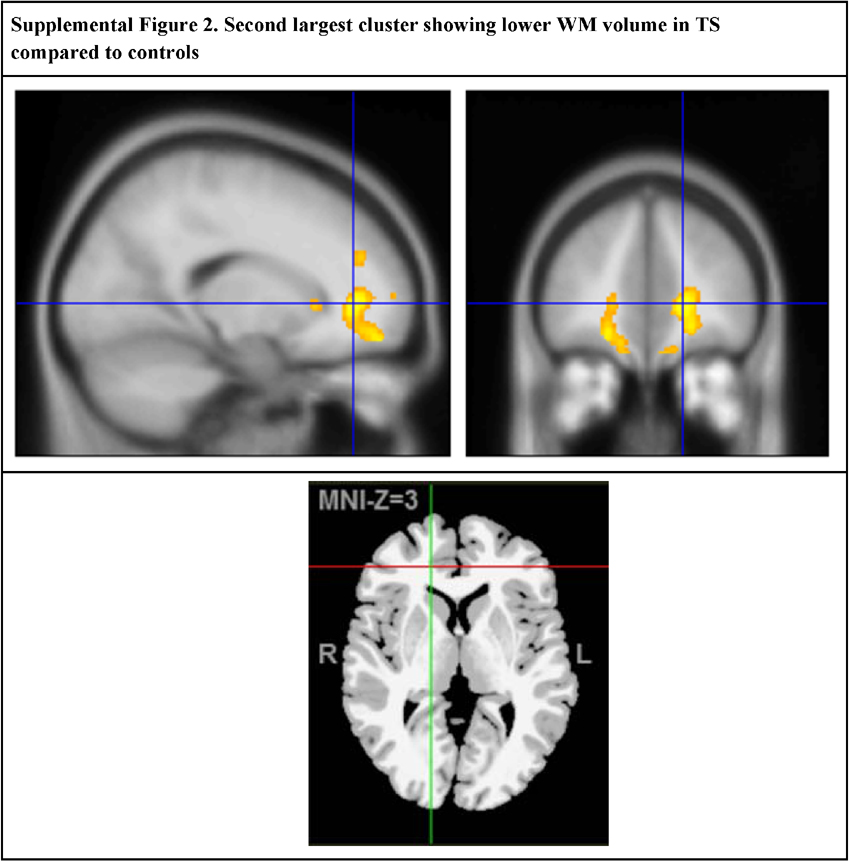

Supplement: Supplementary file 2 — Supplementary Figure 2 (JPG 225 kb) [file 41380_2017_BFmp2016194_MOESM363_ESM.jpg]

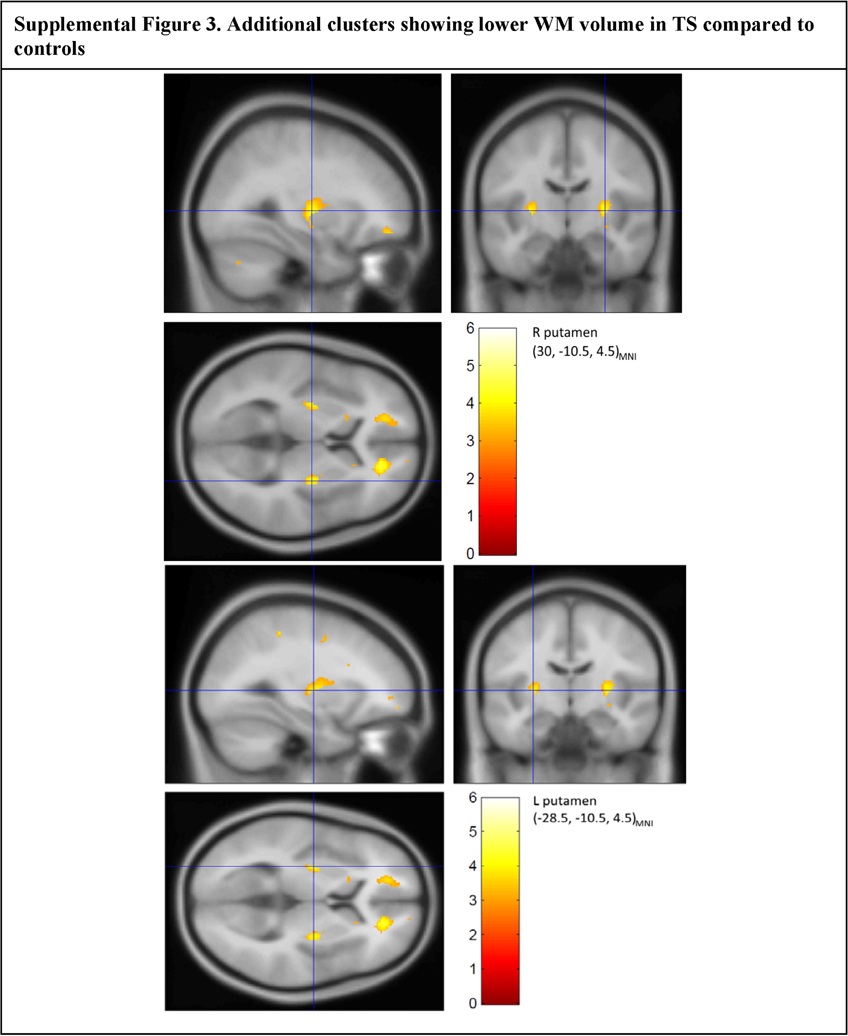

Supplement: Supplementary file 3 — Supplementary Figure 3 (JPG 263 kb) [file 41380_2017_BFmp2016194_MOESM364_ESM.jpg]

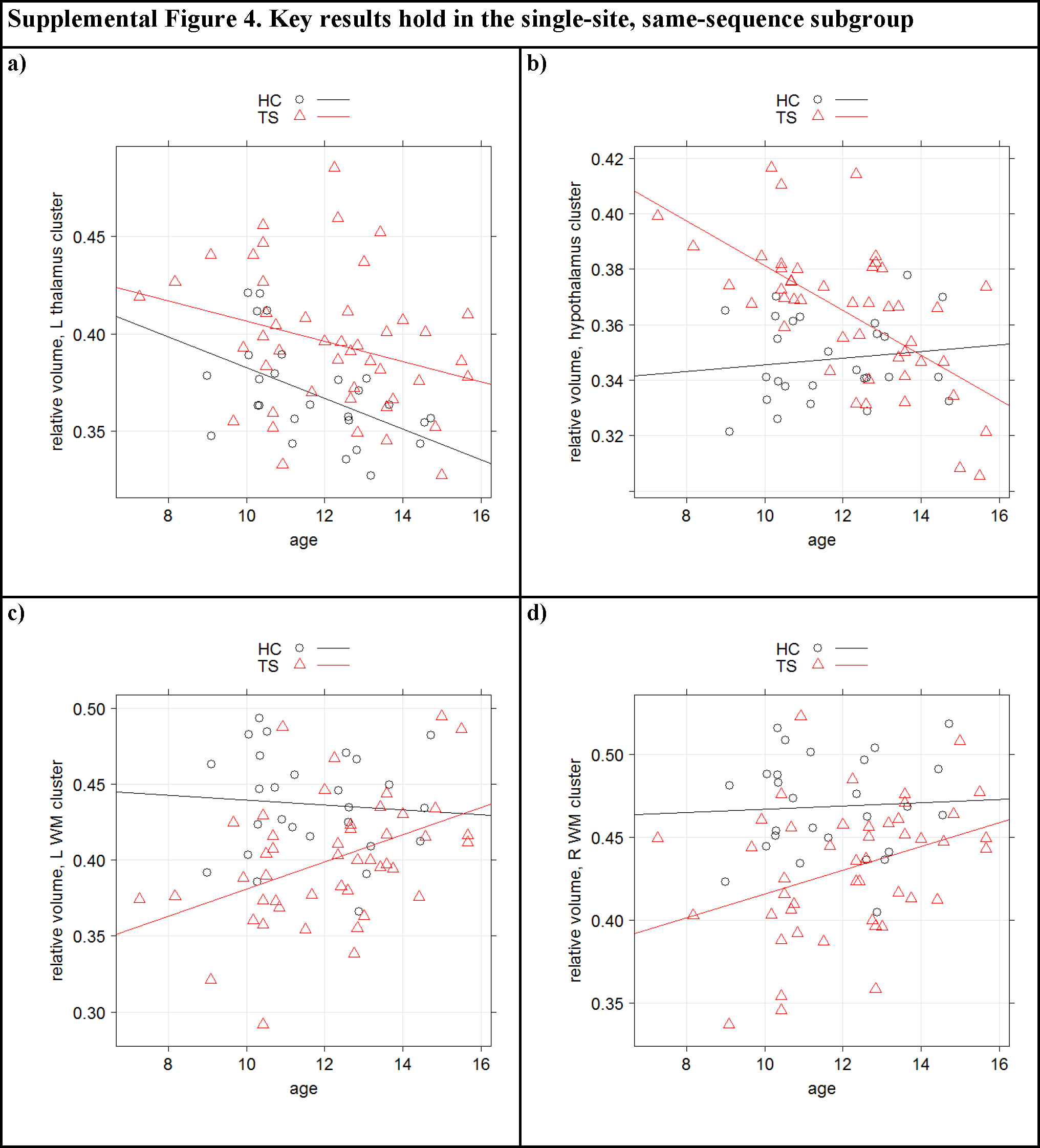

Supplement: Supplementary file 4 — Supplementary Figure 4 (JPG 1086 kb) [file 41380_2017_BFmp2016194_MOESM365_ESM.jpg]

## Slide 1
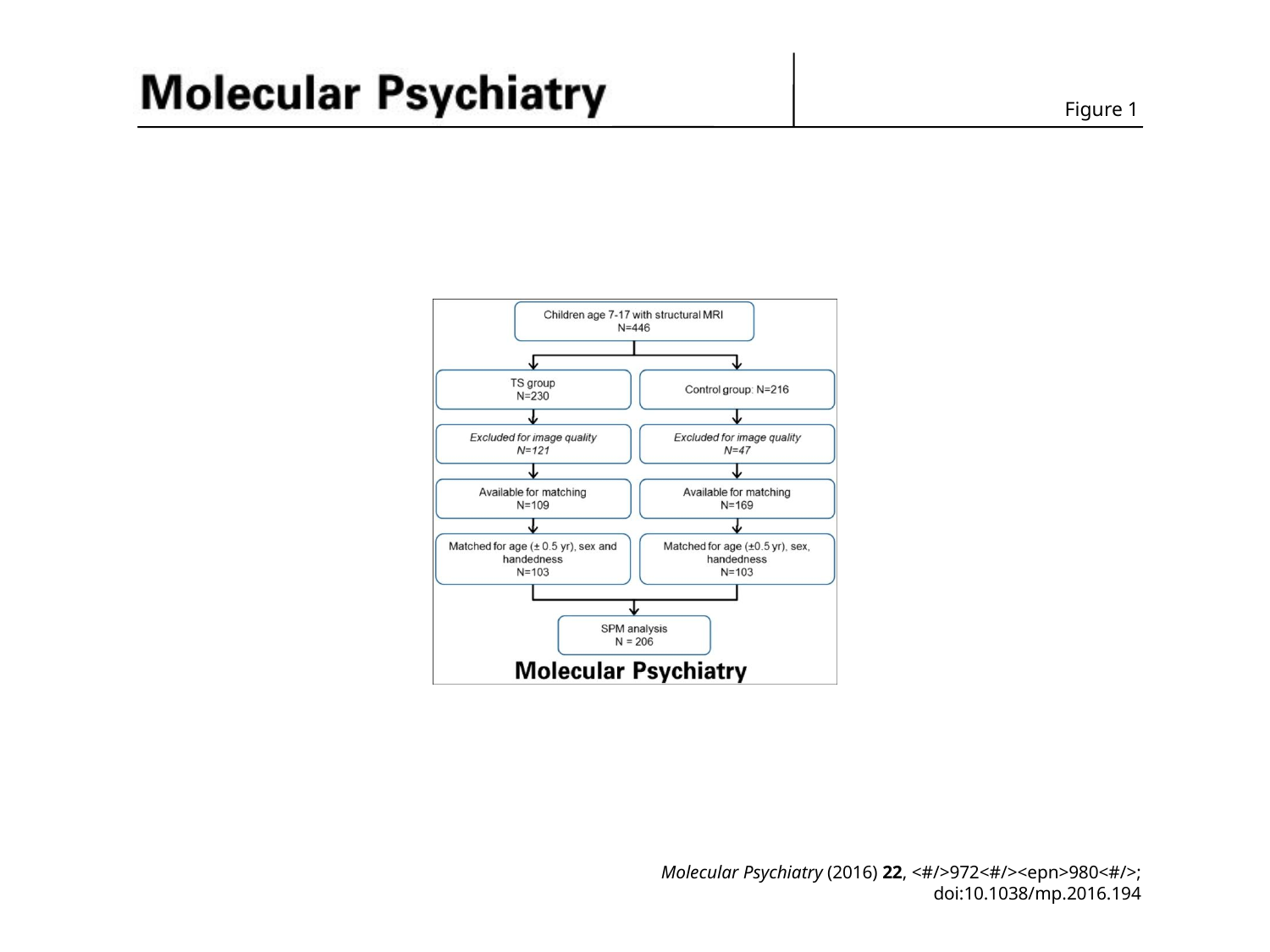

Figure 1
Molecular Psychiatry (2016) 22, <#/>972<#/><epn>980<#/>;
doi:10.1038/mp.2016.194

Supplement: Supplementary file 6 — PowerPoint slide for Fig. 1 [file 41380_2017_BFmp2016194_MOESM359_ESM.ppt]

## Slide 1
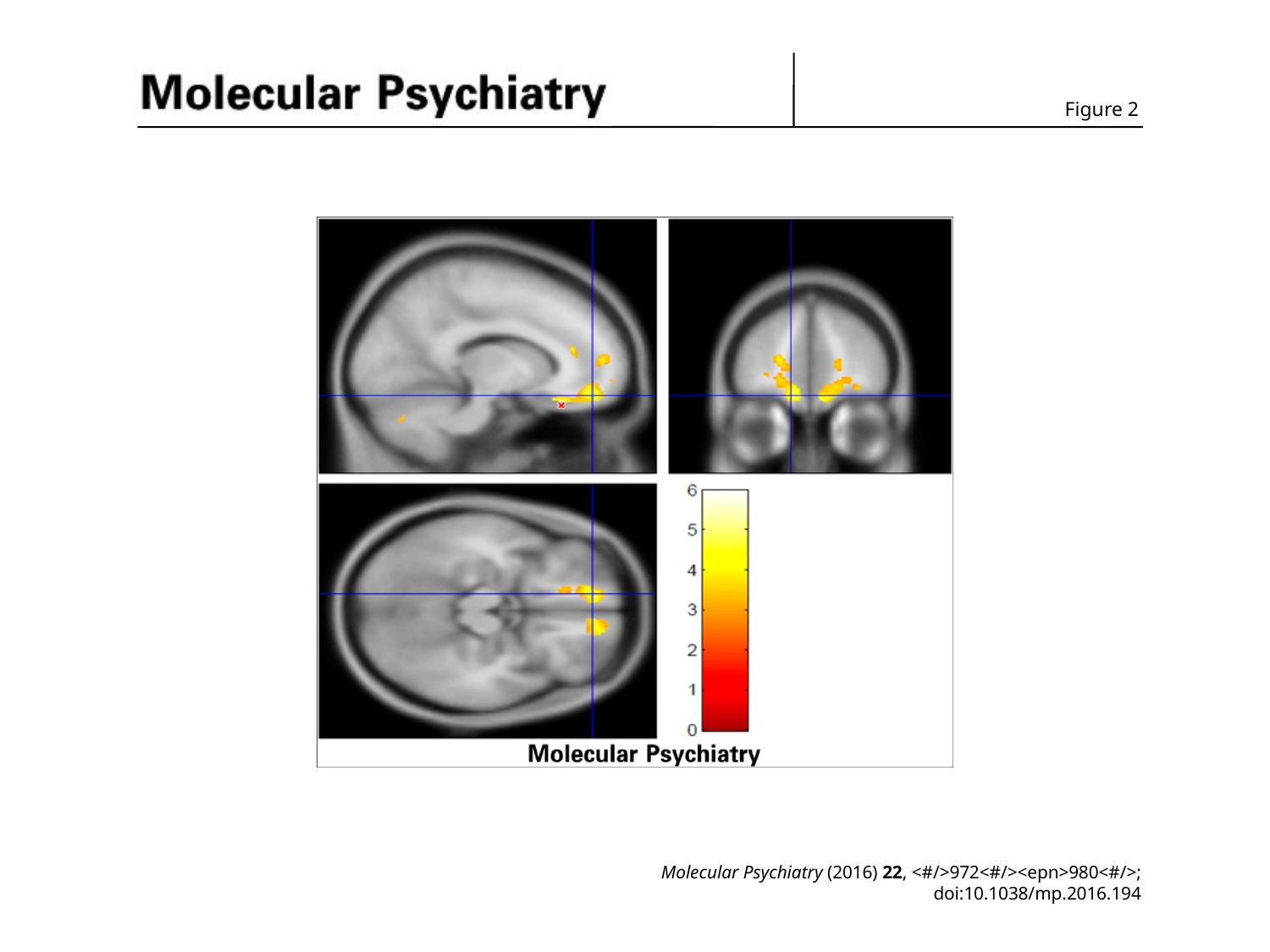

Figure 2
Molecular Psychiatry (2016) 22, <#/>972<#/><epn>980<#/>;
doi:10.1038/mp.2016.194

Supplement: Supplementary file 7 — PowerPoint slide for Fig. 2 [file 41380_2017_BFmp2016194_MOESM360_ESM.ppt]

## Slide 1
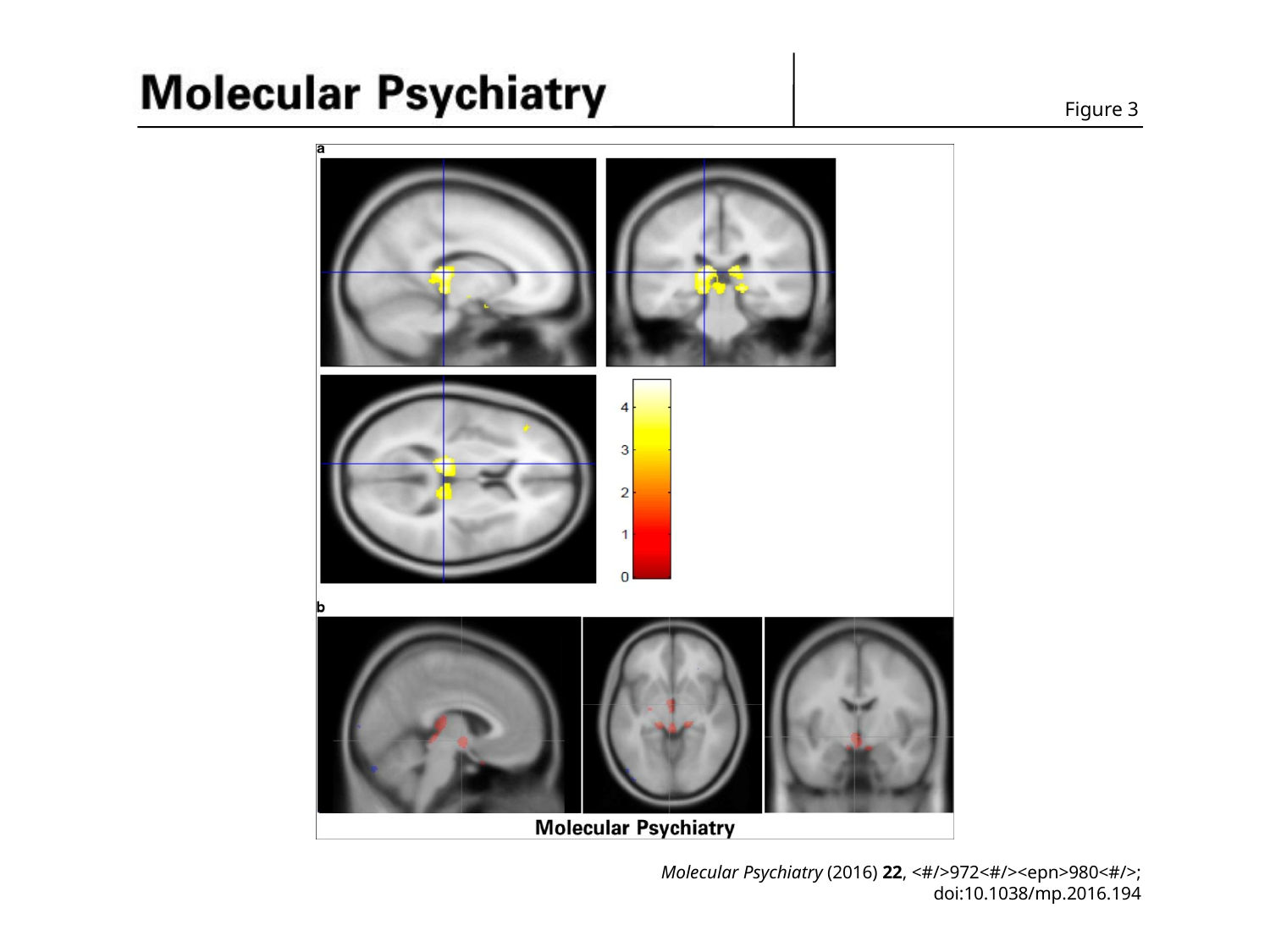

Figure 3
Molecular Psychiatry (2016) 22, <#/>972<#/><epn>980<#/>;
doi:10.1038/mp.2016.194

Supplement: Supplementary file 8 — PowerPoint slide for Fig. 3 [file 41380_2017_BFmp2016194_MOESM361_ESM.ppt]
