# Supplementary material for: Brain structure in pediatric Tourette syndrome
Source: Mol Psychiatry. 2016 Oct 25;22(7):972–80. doi: 10.1038/mp.2016.194 (PMC5405013; doi:10.1038/mp.2016.194)
Supplement: Supplementary file 5 — Supplementary Table 1 (DOCX 92 kb) [file 41380_2017_BFmp2016194_MOESM366_ESM.docx]

| **Supplemental Table 1. Image collection specifications** | | | | | |
| --- | --- | --- | --- | --- | --- |
|  | **Site** | **Scanner** | **Sequence parameters** | **TSN*** | **TSC*** |
| 1 | NYU | Allegra | *tfl3d1_ns; 3.0T, TR = 2530 ms, TE = 3.25 ms, TI = 1100 ms, flip angle = 7°, voxel size = 1.0mm × 1.0mm within-plane, 128 sagittal slices acquired 1.33mm apart | 8 | 21 |
| 2 | WUSM | Vision | mpr_ns_t1_4b195; Siemens 1.5T MAGNETOM Vision, sagittal magnetization-prepared rapid gradient echo (MP-RAGE), TR = 9.7 ms, TE = 4 ms, flip angle = 10°, voxel size = 1.0 mm × 1.0 mm × 1.25 mm. | 11 | 2 |
| 3 | WUSM | Trio,  East Building | mprage, 256×256×176 voxels, *tfl3d1_ns, 1.0 × 1.0 × 1.0mm, TR 2400, TE 3.12, TI 1000, flip angle = 8° | 46 | 27 |
| 4 | WUSM | Trio, CCIR | same | 0 | 39 |
| 5 | WUSM | Vision | MPRAGE, voxels 1.0 × 1.0 × 1.25 mm, acquisition time about 6.5 minutes | 2 | 2 |
| 6 | KKI | Intera | T1TFE; 3.0T, TR = 8.07 sec, TE = 3.689 ms, flip angle = 8°, 1.0mm × 1.0mm within-plane resolution, 200 coronal slices acquired 1.0mm apart | 4 | 12 |
| 7 | UCLA | Allegra | *tfl3d1; 3.0T, TR = 2300 ms, TE = 2.1 ms, TI = 1100 ms, flip angle = 8°, 1.33mm × 1.33mm within-plane resolution, 160 sagittal slices acquired 1.0mm apart | 13 | 0 |
| 8 | WUSM | Trio | t1_mpr_ns_sag_ipat; tfl3d1_ns; 3.0T, TR = 2200 ms, TE = 2.34 ms, TI = 1000 ms, flip angle = 7°, voxel size = 1.0mm × 1.0mm within-plane resolution, 128 sagittal slices acquired 1.33mm apart, GRAPPA on | 19 | 0 |
| *TSN: Number of TS subjects in this analysis from the specified sequence. TSC: Number of control subjects  in this analysis from the specified sequence. NYU = New York University. WUSM = Washing University  School of Medicine. KKI = Kennedy Krieger Institute at Johns Hopkins University School of Medicine.  UCLA = University of California, Los Angeles. | | | | | |
